# A plural role for lipids in motor neuron diseases: energy, signaling and structure

**DOI:** 10.3389/fncel.2014.00025

**Published:** 2014-02-20

**Authors:** Florent Schmitt, Ghulam Hussain, Luc Dupuis, Jean-Philippe Loeffler, Alexandre Henriques

**Affiliations:** ^1^Mécanismes Centraux et Périphériques de la Neurodégénerescence, INSERM U1118Strasbourg, France; ^2^UMRS1118, Fédération de Médecine Translationnelle de StrasbourgUniversité de Strasbourg, France

**Keywords:** ALS, motor neuron, lipid, metabolism, SMA, SBMA

## Abstract

Motor neuron diseases (MNDs) are characterized by selective death of motor neurons and include mainly adult-onset amyotrophic lateral sclerosis (ALS) and spinal muscular atrophy (SMA). Neurodegeneration is not the single pathogenic event occurring during disease progression. There are multiple lines of evidence for the existence of defects in lipid metabolism at peripheral level. For instance, hypermetabolism is well characterized in ALS, and dyslipidemia correlates with better prognosis in patients. Lipid metabolism plays also a role in other MNDs. In SMA, misuse of lipids as energetic nutrients is described in patients and in related animal models. The composition of structural lipids in the central nervous system is modified, with repercussion on membrane fluidity and on cell signaling mediated by bioactive lipids. Here, we review the main epidemiologic and mechanistic findings that link alterations of lipid metabolism and motor neuron degeneration, and we discuss the rationale of targeting these modifications for therapeutic management of MNDs.

## Introduction

Motor neuron diseases (MNDs) are a group of incurable neurological disorders caused by the selective degeneration of motor neurons. Amyotrophic lateral sclerosis (ALS) is the most representative MNDs among adults with a incidence rate of 2–3 per 100,000 (Brooks et al., 2000). It is characterized by progressive muscle weakness and atrophy, loss of upper and lower motor neurons and death ensuing 3–5 years after diagnosis. Majority of ALS patients are of sporadic origin with unclear ethiopathology. Several mutations are associated with ALS, in particular in genes encoding superoxide dismutase 1 (*SOD-1*), TAR DNA binding protein of 43-kDa (*TDP-43*), fused in sarcoma *(FUS*) and chromosome 9 open reading frame 72 (*C9ORF72*) (Rosen et al., 1993; Mackenzie et al., 2007; Deng et al., 2010; Laaksovirta et al., 2010; Shatunov et al., 2010). Thus, several transgenic mouse models overexpressing various mutant genes have been developed, and the SOD1 model, overexpressing a mutated form of SOD1 gene, is the most studied in ALS (Ripps et al., 1995). Spinal muscular atrophy (SMA) is a genetic autosomal and recessive neuromuscular disease, caused by loss of functional survival motor neuron (SMN) gene 1. Patients suffer from degeneration of spinal motor neurons, muscle weakness leading to atrophy. Disease severity ranges from severe, with death of patients before the age of 10, to mild with moderate symptoms with no alteration of life expectancy. Spinal bulbar muscular atrophy (SBMA), also known as Kennedy's disease, is an X-linked recessive disease caused by a CAG-repeat expansion in the gene coding for the androgen receptor, leading to a poly-Q repeat expansion in the protein (Brooks and Fischbeck, 1995; Fischbeck et al., 1999). The disease affects mainly males, even though it has been described also in female patients. SBMA is characterized by progressive muscle atrophy and degeneration of lower motor neurons in the brain stem and spinal cord. Similarly to ALS, mouse models have been developed to study SMA and SBMA (Katsuno et al., 2003; Bebee et al., 2012).

Along with neuronal degeneration, several alterations of lipid metabolism are found in these diseases. Here, we review the role of lipids in MNDs, with a special attention on energy homeostasis, cell signaling and structure. We further discuss the rationale of targeting lipid metabolism for therapeutic management of MNDs.

## Energetic alterations in amyotrophic lateral sclerosis

Multiple previously unrecognized phenotypes occur in ALS patients, in particular, the unbalance between food intake and energy (Braun et al., 2012; Muscaritoli et al., 2012). ALS patients often present with dyslipidemia, reduced body mass and increased resting energy expenditure (Table 1, Desport et al., 2001; Funalot et al., 2009; Dupuis et al., 2011). Energetic alterations are similarly found in transgenic animal models of ALS, the SOD1 mice. These mice are leaner than controls, hypermetabolic, hypolipidemic and present increased fatty acid uptake in muscles (Table 2, Dupuis et al., 2004; Fergani et al., 2007; Kim et al., 2011). Several lines of evidence point to lipid metabolism alterations being crucial for ALS progression. A first incidental event shed some light on the subject. During 2 years, a group of scientists remain in an isolated environment, and were submitted to long term caloric restriction (Walford et al., 2002). From eight members of the group, one died from ALS and another one developed progressive gait impairment and motor neuron degeneration. Epidemiological studies went beyond this case study and documented an association between nutrition and the risk of developing ALS. First, a case control survey made in Japan in 2007 was conducted with the aim to study pre-illness nutritional habits of ALS patients. The authors identified that high carbohydrate and low fat intakes are associated with higher ALS risk (Table 1, Okamoto et al., 2007). More recently, another epidemiologic study focused on anthropometric characteristics of the general population with a follow up over 10 years. The investigators reached the conclusion that high fat content reduces the risk of developing ALS (Gallo et al., 2013). These clinical data are consistent with the reduced overall survival of SOD1 mice under caloric restriction (Table 2) (Hamadeh et al., 2005).

**Table 1:** **Altered energetic metabolism in ALS patients**.

**Items**	**Cohort size (ALS patients)**	**Outcomes**	**References**
**BASAL METABOLISM IN ALS**			
	*N* = 62	ALS patients are hypermetabolic.	Desport et al., 2001
	*N* = 44	ALS patients are hypermetabolic.	Funalot et al., 2009
**EPIDEMIOLOGIC AND RISKS TO ALS**			
	*N* = 153	High carbohydrates and low fat intakes increase ALS risk.	Okamoto et al., 2007
	*N* = 222	High prediagnostic body fat is associated with a decreased risk of ALS mortality.	Gallo et al., 2013
**LIPIDS AND PROGNOSIS IN ALS**			
	*N* = 369	High LDL/HDL ratio correlates to longer survival.	Dupuis et al., 2008
	*N* = 658	Hyperlipidemia does not correlate to longer survival.	Chio et al., 2009
	*N* = 658	Low level LDL/HDL ratio correlates to respiratory dysfunction.	Chio et al., 2009
	*N* = 285	Low BMI correlates to faster decline.	Jawaid et al., 2010
	*N* = 92	Poor nutritional status is associated with higher mortality.	Marin et al., 2011
	*N* = 488	High triglycerides or cholesterol correlates to longer survival.	Dorst et al., 2011
	*N* = 427	High BMI correlates to longer survival.	Paganoni et al., 2011
	*N* = 77	Fast reduction of BMI predicts faster decline.	Shimizu et al., 2012
	*N* = 150	High BMI correlate to slower ALSFRS score decline.	Reich-Slotky et al., 2013
	*N* = 62	High subcutaneous fat positively correlate to survival.	Lindauer et al., 2013

**Table 2:** **Altered energetic metabolism in mouse models of MNDs**.

**Model**	**Cohort size**	**Conclusion**	**References**
**ALS**			
SOD1 G93A mice	*N* = 8	Altered composition of lipids in spinal cord.	Cutler et al., 2002
SOD1 G86R and G93A mice	*N* = 7–8	Dramatic defect in energy homeostasis, hypermetabolism mainly of muscular origin.	Dupuis et al., 2004
	*N* = 13	High fat diet delays disease onset and extent survival.	Dupuis et al., 2004
SOD G93A mice	*N* = 27	The ketogenic diet protects against motor neuron death.	Zhao et al., 2006
SOD1 G86R and G93A mice	*N* = 10–15	Hypolipidemia is found in SOD1 mice.	Fergani et al., 2007
SOD1 G93A mice	*N* = 49 females	Caloric restriction shortens lifespan through an increase in lipid peroxidation, inflammation and apoptosis.	Patel et al., 2010
	*N* = 31 males		
SOD1 G93A mice	*N* = 30 males	Hypolipidemia is present at the presymptomatic stage of disease.	Kim et al., 2011
SOD1 G93A mice	*N* = males	Medium chain triglycerides protect motoneurons survival but does not extent survival.	Zhao et al., 2012
**SMA**			
SMN1 deficient mice	*N* = 30 males	Hypolipidemia is present at the pre-symptomatic stage of disease.	Butchbach et al., 2010

Conversely, increased energy intake is beneficial for SOD1 mice. The first evidence emerged in 2004 from our laboratory, when we fed SOD1 mice with a diet enriched in lipids. The treatment restored normal body mass and adiposity, delayed disease onset and motor neuron degeneration, and life expectancy was extended by 20% (Dupuis et al., 2004). The beneficial effects of high fat diet, or ketogenic diet, for SOD1 mice were confirmed by other groups (Table 2, Mattson et al., 2007; Zhao et al., 2006, 2012). In 2008, we aimed to study the link between lipids and disease progression, by quantifying circulating lipids in ALS patients. We found that dyslipidemia, defined by high LDL/HDL ratio, was a characteristic of the ALS group (Dupuis et al., 2008), and this dyslipidemia positively correlated with longer survival, increased by 13 months in the group of ALS patients with higher LDL/HDL ratio. Since then, others reported that either hyperlipidemia or high body mass index is a strong prognostic factor for survival (Dorst et al., 2011; Paganoni et al., 2011; Shimizu et al., 2012; Reich-Slotky et al., 2013). For instance, the median life expectancy was higher than 14 months in patients with high serum triglyceride levels (Dorst et al., 2011). Last, it was recently observed that subcutaneous fat positively correlated with survival of ALS patients (Lindauer et al., 2013). Conversely, lower LDL/HDL ratio has been linked to respiratory impairments (Chio et al., 2009), fast loss of BMI was associated with faster decline (Jawaid et al., 2010) and poor nutritional status is a negative prognosis factor (Marin et al., 2011).

These reports claim for a positive correlation between high circulating lipid levels of lipids or high fat mass and prognosis of ALS patients. The mechanisms in place deserve further investigation, to understand how peripheral lipids interfere with disease progression and how lipids can preserve motor axis integrity.

## Energetic substrates and motor units

The motor unit is an anatomical structure responsible for the control of muscle contraction and its destruction represents the first detectable event in ALS (Dupuis and Loeffler, 2009). The motor units are composed of different sorts of spinal motor neurons and muscles fibers, depending on their activity and energetic capacities. Fast fatigable motor units are composed of large alpha-motor neurons and glycolytic muscle fibers that burn preferentially glucose to exert heavy force on a short period. Conversely, slow motor units correspond to small alpha-motor neurons and oxidative muscle fibers, which store and use preferentially fatty acids to produce less intense but constant strength. Large alpha motor neurons are the first to degenerate in ALS models (Pun et al., 2006; Hegedus et al., 2007), and fast-twitch motor units are preferentially affected in both ALS patients and mouse models (Schmied et al., 1999; Atkin et al., 2005; Gordon et al., 2010). Stimulation of motor units via regular training was proposed to maintain and strengthen motor function in ALS (Table 3). Clinical investigations reported benefit for ALS patients whom followed moderate and mainly aerobic exercise program, which use lipids as energy source (de Almeida et al., 2012). In particular, specific training of the diaphragma preserved respiratory functions in ALS patients (Mahajan et al., 2012). It should be noted that these studies were not randomized and suffer from the small size of the cohorts. Moreover, higher release of reactive oxygen species during exercise was reported (Siciliano et al., 2002) and suggest that design for muscular training in ALS patients should be considered with caution (Table 3).

**Table 3:** **Effect of exercise in ALS**.

**Cohort**	**Outcomes**	**References**
**SOD1 MICE G93A**		
	High frequency and amplitude training support in motor neuron survival.	Deforges et al., 2009
	Moderate frequency and amplitude training support motor neurons survival.	Carreras et al., 2010
	Benefits of training might come from the enrichment of the environment.	Gerber et al., 2012
**ALS PATIENTS**		
	Regular moderate physical exercise should be recommended.	Drory et al., 2001
	Higher release of ROS during exercise suggests that design of training should be considered with caution.	Siciliano et al., 2002
	Benefits of moderate aerobic exercise.	Bello-Haas et al., 2007

In an animal model of ALS, effects of high frequency and high amplitude training were compared to moderate and endurance training. SOD1 mice were subjected either to swimming-based training, targeting glycolytic motor units, or to running-based training, targeting oxidative motor units (Deforges et al., 2009). After training, the group of “swimmer” mice presented with benefits in terms of counts of motor neurons and survival, when compared to proper control. On the contrary, the “running”-trained mice presented a similar disease course compared to sedentary mice. Effect of training on SOD1 mice is not fully clarified. Others reported claimed that moderate exercise alone can improve outcomes in SOD1 mice (Carreras et al., 2010), and most importantly, Gerber and colleagues recently demonstrated that benefits of training in mice might come from the enrichment of the environment rather than mobilization of muscle fibers (Gerber et al., 2012). Therefore, the benefit after different types of training for SOD1 mice targeting different pool of motor units remains to be clarified. Currently, the effects of aerobic versus anaerobic exercises on vital capacity and muscular strength are under clinical investigations (Table 4, NCT01650818, NCT01521728). Additional work on this topic is required to understand the impact of different exercises, using different energetic sources, on motor units in ALS patients, and why glycolytic motor units are preferentially affected.

**Table 4:** **Ongoing clinical trials in ALS**.

**Items**	**Clinical trial ID**	**Status**	**Study type**	**Intervention**	**Estimated enrollment**	**Study**
**EXERCISE**						
	NCT01650818	Recruiting	Interventional	Endurance training	40	Aerobic exercise training in ALS
	NCT01521728	Recruiting	Interventional	Resistance exercise	60	Trial of resistance and endurance exercise in ALS
**DIETARY SUPPLEMENT**						
	NCT00983983	Completed	Interventional	Oxepa	30	High fat/high calorie trial in ALS
	NCT01016522	Terminated	Interventional	KetoCal	NA	Safety and tolerability of the ketogenic diet in ALS
**BASAL METABOLISM**						
	NCT00714220	Recruiting	Observational	/	150	Quantitative measurement of nutritional substrate utilization in patients with ALS
	NCT01592084	Completed	Observational	/	267	Hyperlipidemia and statin therapy in ALS
**PHARMACOLOGY**						
	NCT00690118	Terminated	Interventional	Pioglitazone (45 mg/day)	219	Study of pioglitazone in patients with Amyotrophic Lateral Sclerosis
	NCT00876772	Unknown	Interventional	Olanzapine (10 mg/day)	40	Olanzapine for the treatment of appetite loss in Amyotrophic Lateral Sclerosis (ALS)

Clinical trial IDs refer to the current nomenclature used at clinicaltrial.gov.

## Energy, metabolism, and mitochondria

Taken separately, the two extremities of the motor units, the muscles and the motor neurons, show abnormal lipid metabolism. Indeed, early in the disease course, glycolytic muscles of SOD1 mice switch toward an oxidative phenotype, presumably due to the loss of their connection to large motor neurons and subsequent reinnervation by “slow” motor neurons (Sharp et al., 2005). The selective vulnerability of large motor neurons is potentially due to higher energetic needs that could either be not fulfilled or a source of oxidative stress. In case of higher needs, neurons can use ketone bodies as energetic substrate when glucose level becomes low (Guzman and Blazquez, 2004; LaManna et al., 2009). In this situation, astrocytes will use lipid to provide ketone bodies to neurons, and potentially motorneurons in ALS (Yi et al., 2011). Interestingly, medium chain triglycerides, precursors of ketone bodies, preserve motor functions and promote motorneuron survival in SOD1 mice through the enhancement of oxidative metabolism (Table 2, Zhao et al., 2012).

Moreover two recent studies have shown abnormally enhanced levels of ketone bodies, released by the breakdown of fatty acids, in the cerebrospinal fluid of ALS patients (Blasco et al., 2010; Kumar et al., 2010) that could account for an altered lipid beta oxidation in the CNS of ALS patients.

Higher energetic needs have been documented in muscle of SOD1 mice (Dupuis et al., 2004). In the animal model, the metabolic shift in muscle fibers (glycolytic to oxidative) could explain the pronounced appetence for fatty acids in SOD1 muscles, as well as the changes in the expression profile of genes involved in lipid metabolism (Fergani et al., 2007; Gonzalez de Aguilar et al., 2008; Thau et al., 2012). Interestingly, the predominance of oxidative metabolism goes along with deficiency in oxidative mitochondrial chain function, in particular in the muscles of ALS patients (Echaniz-Laguna et al., 2002; Dupuis et al., 2003; Crugnola et al., 2010). Mitochondria bioenergetic functions are impaired, as shown by Zhou and colleagues, whom described the presence of defective mitochondria in mass near the neuromuscular junctions that may contribute to the progression of muscle atrophy in ALS (Zhou et al., 2010). The origin of mitochondrial defect is under investigation. Several potential pathways are proposed to explain the shift in metabolism and the alterations of mitochondrial functions, and they concern the tuning of metabolic pathways.

Mitochondrial biogenesis and functions are orchestrated in part by peroxisome proliferator-activated receptor gamma coactivator (PGC)-1 alpha (Lin et al., 2002, 2005; Handschin, 2010). In ALS, the implication of PGC-1 alpha has been recently highlighted, as its expression is diminished in the muscles of patients and SOD1 mice (Thau et al., 2012). The downregulation of PGC-1 alpha triggers modification of lipid metabolism, and impacts the use of fatty acids (Barroso et al., 2011). Interestingly, we have very recently shown that deficiency in PGC1-alpha leads to hasten disease progression in the males of a mouse model of ALS (Eschbach et al., 2013), strengthening the relation between lipid metabolism alterations and disease progression, at least in SOD1 mice. When Da Cruz and collaborators overexpressed PGC1-alpha selectively in the muscles of SOD1 mice, they observed improved locomotor activity and reduced muscle atrophy, but no effects on the overall survival of this mouse line (Da Cruz et al., 2012). Their results suggest that improving muscle activity and reducing atrophy through increased PGC1-alpha could be used as a palliative treatment in ALS. In addition, the overexpression of PGC1-alpha selectively in the central nervous system (CNS) of SOD1 mice restored the activity of mitochondrial complexes in the spinal cord, supported motor functions and enhances survival by 8% (Zhao et al., 2011). Moreover sirtuin 3, a downstream target of PGC-1 alpha, protects neurons *in vitro* against SOD1 G85R toxicity (Song et al., 2013). General mitochondrial activity, including mitochondrial proliferation, is impaired in ALS and represents a promising therapeutic target for ALS as recently discussed (Cozzolino et al., 2013; Dupuis, 2013; Pasinetti et al., 2013).

Stearoyl-Coa desaturase 1 (SCD-1) is a key enzyme for the regulation of fatty acid metabolism, and it can impact fatty acid oxidation taking place in mitochondria. SCD-1 introduce a double bond in the carbon chain of saturated, to generated mono-unsaturated fatty acids that are more prone to be stored in fat tissues. We have recently reported a downregulation of SCD-1 in the muscle of SOD1 mice (Hussain et al., 2013), and in a subpopulation of ALS patients (Pradat et al., 2011). The function of SCD-1 is associated to regulation of energetic metabolism, and most particularly the management of lipid reserves. Downregulation of SCD-1 is known to trigger increased expression of genes involved in the beta-oxidation of fatty acids, increased energy expenditure and reduced fat storage, a metabolic phenotype exhibited by SOD1 mice (Ntambi et al., 2002; Dupuis et al., 2004). We aimed to study the impact of a low SCD-1 activity for the motor function. We recently described that knock-out mice for SCD-1, and non-transgenic mice treated with a SCD-1 inhibitor, present improved nerve regeneration after peripheral nerve injury (Hussain et al., 2013). Moreover, the products of SCD-1, the mono-unsaturated fatty acids, favor cytotoxic SOD-1 aggregation (Kim et al., 2005), and the accumulation of toxic lipid species such as ceramide (Dobrzyn et al., 2005), suggesting that loss of SCD-1 activity could lower cytotoxicity in ALS. Further work is needed to understand the link between loss of SCD-1 activity and benefits for the motor units, especially in ALS. Aside from its role in energetic metabolism, SCD-1 is additionally required in the synthesis of more complex lipids, including phospholipids. Alterations in lipid metabolism will have repercussion not only on the energy homeostasis, but also on a wide range of cellular functions, including membrane fluidity and signaling.

## A role for lipids beside energetic metabolism

Lipids play a critical role in the structure of the central and peripheral nervous systems in particular at the cell membrane level. They control membrane fluidity, improve transmission of electrical signals and stabilize synapses.

### Membrane fluidity

Basic cellular functions depend on the composition in lipids of plasmatic membranes. Enrichment of sphingolipids and cholesterol, as well as content in polyunsaturated fatty acids (PUFA), directly determines membrane fluidity and movement of membrane proteins in lipid rafts (Xu et al., 2001; Lang, 2007; Lingwood and Simons, 2010). Although the level of these lipids is altered in ALS patients and SOD1 mice, membrane fluidity *per se* has not been extensively investigated. One recent study described loss of membrane fluidity in the SOD1 mice at disease onset (Miana-Mena et al., 2011) presumably due to oxidative stress and lipid peroxidation. Membrane phospholipids in the CNS are rich in PUFA and in particular of docosahexaenoic acid (DHA). Interestingly, the profile of fatty acids in the brain cortex and spinal cord of ALS patients revealed an increase of DHA level with potential consequences on membrane fluidity (Ilieva et al., 2007). Changes in the membrane fluidity could affect wide range of cellular functions such as ligand-receptor signal transduction and membrane trafficking (Simons and Vaz, 2004), with consequences on cell functions and survival.

### A direct role for lipids in motorneuron survival?

Back in 2002, the group of Pr. Mattson studied the lipid metabolites present in the spinal cord of both ALS patients and pre-symptomatic SOD1 mice, and reported higher amounts of sphingolipids and cholesterol associated with increased lipid peroxidation (Cutler et al., 2002). These findings are important as these lipid metabolites modulate vital cellular functions in the CNS that may be involved in ALS pathophysiology. Aberrant accumulation of ceramides is commonly seen as being toxic. It mediates neuron death by oxidative stress and apoptosis in animal models and patients of neurodegenerative diseases (Brugg et al., 1996; France-Lanord et al., 1997; Bras et al., 2008; Car et al., 2012; Filippov et al., 2012). Ceramides are precursor molecules at the crossroads of the sphingolipid metabolism and they can be converted into sphingomyelin, ceramide-1-phosphate and gangliosides. Abnormal repartition of gangliosides was described in the CNS of ALS patients and presence of antibodies anti-gangliosides has been described in their serum (Mizutani et al., 2003). Gangliosides are important for axonal function and regeneration, and neuronal survival (Akasako et al., 2011). In the 80's, clinicians initiated half-dozen trials in ALS with injection of gangliosides with the aim to protect the motor units, but lead to no benefit (Bradley, 1984; Hallett et al., 1984). These studies were however underpowered. Sphingomyelin is particularly abundant in the nervous systems and represents another lipid impacting motor neuron survival. Cutlers and colleagues proposed that the increase of sphingomyelin in the spinal cord of ALS patients mediates motor neuron death via oxidative stress (Cutler et al., 2002), and in 2007, another group reported that p75-mediated motor neuron death is stimulated upon sphingomyelin-associated ROS production in an animal model of ALS (Pehar et al., 2007).

### Signaling molecules

In addition to their role in the membrane structure, PUFA also have intrinsic functions on cell signaling, in particular on neuroinflammation and regulation of energetic metabolism. First, PUFA are known to bind to transcription factors, such as liver-X receptor and retinoic-X receptor (Yoshikawa et al., 2002), to stimulate the expression of genes involved in energy homeostasis and dysregulation of their levels could account for the altered metabolism in ALS.

PUFA can be also converted to active molecules. Depending on the location of the unsaturations, PUFA present either anti-inflammatory and neuroprotective effects, for the omega 3 fatty acids, or pro-inflammatory for omega 6 fatty acids (Schmitz and Ecker, 2008). For instance, eicosapentaenoic and arachidonic acids can be oxidized to give rise to prostanglandins or leukotrienes, and the oxidation of DHA produces the neuroprotectin D1, a signaling molecule that promotes beneficial effects on cell survival under stress (Bazan et al., 2011). Prostaglandin E2 (PGE2) is synthetized by cyclooxygenase-2 from the arachidonic acid, an omega 6 fatty acid, to promote inflammation after binding to its receptor. In ALS patients, level of PGE2 is increased in the serum and the cerebrospinal fluid (Ilzecka, 2003). Evidence from animal models also suggests a role for PGE2 in ALS physiopathology. Indeed, the pharmacological inhibition of PGE2 receptor or the silencing of the gene coding for cyclooxygenase-2 can lower neuroinflammation in SOD1 mice, preserve motor functions and extend survival (Pompl et al., 2003; Klivenyi et al., 2004; Liang et al., 2008).

The omega 3 fatty acids can be converted into anti-inflammatory and neuroprotective molecules. Many reports have shown that treatments based on omega 3 fatty acids exert beneficial effects in various animal models of neurodegenerative disease, such as Parkinson's or Alzheimer's diseases (Arsenault et al., 2011; Ozsoy et al., 2011). In a recent study, Michael-Titus and colleagues investigated the neuroprotective effect of eicosapentaenoic acid, an omega 3 fatty acid precursor for DHA, in SOD1 mice. Unexpectedly, the treatment resulted in enhancement of neuroinflammation, faster disease progression and hastened death for SOD1 mice (Yip et al., 2013). One explanation proposed by the authors is the greater susceptibility for PUFA to be peroxidized and therefore toxic (Kanner et al., 1987). Therefore, the increase in DHA of spinal cord from ALS patients could favor lipid peroxidability, and be potentially a toxic factor in ALS (Ilieva et al., 2007).

Taken together, all these results clearly argue for a strong relationship between lipid metabolism and motor neuron degeneration. Most of the findings originate from ALS, as it is the most studied MNDs. However, alterations of lipid metabolism are also present in other MNDs.

## Lipid alterations in other motor neuron diseases

Motor neuron degeneration is present in several diseases that complete the spectrum of MNDs with ALS. The role of lipids in those diseases is poorly understood, although some lines of evidence exist.

Progressive muscular atrophy (PMA) is rare subtype of ALS characterized by loss of lower motor neurons. To our knowledge, only one report described alterations of circulating lipids in two patients with a PMA syndrome (Yao et al., 1983). However, these patients suffer from other neurological disorders making difficult to reach a conclusion.

SBMA is characterized by progressive muscle atrophy and degeneration of lower motor neurons in the brain stem and spinal cord. Role and regulation of lipids have not been accessed in SBMA patients, although androgen receptors are known to modulate lipid metabolism (Singh et al., 2006). Indeed, the analysis of the muscle transcriptome of SBMA mice revealed that several differentially regulated genes relate to lipid metabolism, from an energetic and a structural point of view (Mo et al., 2010). For instance, the phospholipase A2, group VII (PLA_2_g7) is downregulated in the muscles of SBMA mice. PLA_2_g7 degrades phospholipids to release poly-unsaturated fatty acid and, in muscle, it is involved in the differentiation of myoblasts (Xiao et al., 2012) and reduction of adiposity (Rao et al., 2006). Ddit4l is another example of genes deregulated in SBMA mice. Ddit4l, also known as REDD2, is involved in muscle growth via stimulation of the IGF1/mTOR pathway and muscle fiber switch from oxidative to glycolytic metabolism (Pisani et al., 2005; Miyazaki and Esser, 2009; Mo et al., 2010).

SMA is a progressive, recessively inherited, neuromuscular disease. SMA is characterized by weakness and muscle atrophy due to loss of spinal cord motor neurons. Similarly to ALS, metabolic abnormality is part of pathophysiology of SMA. In SMA, an initial study reported normal body mass index in a mixed population of SMA patients with severe to moderate symptoms. However, in this cohort, the authors described a reduction in fat-free mass but an increase in total fat mass (Sproule et al., 2009). A second study, focusing only on the most severe form of the disease, described a lower calorie intake than the recommended dietary allowance in SMA patients, associated to higher fat mass (Poruk et al., 2012). These alterations potentially participate to failure to thrive described in SMA patients. The reason why SMA patients have high fat mass despite low caloric intake is unclear but strengthen the role of metabolism of lipids in the disease. Indeed, a misuse of lipids has been documented in SMA patients with various symptom severities, with an impaired mitochondrial fatty acid beta-oxidation and a loss of free circulating carnitine, arguing for impairment of fatty acid entry into mitochondria (Tein et al., 1995). Later, the same group of clinicians described fatty acid abnormalities in SMA, such as high dicarboxylic acid to ketone ratio in the plasma when fasting, or low C12:C14 fatty acid ratio, that further decreased within disease duration. These two parameters are strong markers for fatty acid beta oxidation defects (Crawford et al., 1999). The authors concluded that fatty acid abnormality in severe SMA is primary and general defect, directly caused by the loss of the SMN function.

Additionally, in a transgenic animal model of SMA, deficient for the SMN gene, pups survive longer when mothers were fed with a diet enriched in lipid content (Table 2). The treatment also corrected motor dysfunction despite lack of effect on motor neuron survival (Butchbach et al., 2010). Recently, a clinical trial investigated the effect of a combined treatment of valproic acid and L-carnitine in SMA patients, however, no to limited positive effects have been reported in ambulatory and non-ambulatory SMA patients (Swoboda et al., 2010; Kissel et al., 2011). However, further work is required to assess the role of lipids in SMA and their therapeutic potential.

## Paths to explore

Clinical studies and basic research undoubtedly demonstrate a particular connection between lipid metabolism and ALS (Table 4). Contrary to that observed in other neurological diseases, such as Alzheimer's and Parkinson's diseases, or even ageing (Maswood et al., 2004; Patel et al., 2005), high level of lipids positively correlates to better prognosis in ALS. Preclinical data and clinical studies clearly show that dietary lipid supplementation is a promising strategy to treat ALS, although there is no extensive clinical research at this level.

We are aware of only three clinical trials based on nutritional intervention. In a recently published article, Dorst and colleagues aimed to stop weight loss in ALS patients with high caloric diets, either based on fats or carbohydrates. Both interventions were able to stabilize weight loss although the effect was larger in the high-fat diet group (Dorst et al., 2013). Another trial aims to correct malnutrition with high fat dietary supplement in ALS patients (NCT00983983). The second ongoing trial is a phase III that use high ketogenic supplementation (high fat and low carbohydrate) in ALS patients fed through a gastrostomy tube (NCT01016522). These studies are dedicated to the prevention of malnutrition in ALS patients, and are not designed to identify benefit on prognosis after high fat diet. In parallel, how patients on and off respiratory support use diverse nutritional substrates (e.g., lipids) is under investigation (NCT00714220), as well as the influence of lipid lowering therapy in ALS (NCT01592084). There are two additional trials based on pharmacological modulation of energetic metabolism, using pioglitazone or olanzapine. Pioglitazone is an anti-diabetic drug known to manage energetic metabolism and to lower level of circulating lipids in patients suffering from metabolic disorders. This phase II clinical trial has been stopped after interim analysis that revealed no benefit after treatment on primary and secondary outcomes (Dupuis et al., 2012, NCT00690118). A phase II/III using olanzapine is currently ongoing. Olanzapine is a neuroleptic drug with metabolic side effects, and investigators treated ALS patients with olanzapine, based on the weight-increasing effect of the drug (NCT00876772). Study results have not been released yet. The results of these trials will certainly help to determine whether modulation of lipids, though supplementation or pharmacology, is a feasible and rational treatment for ALS, and potentially for other MNDs.

As discussed in this review, the role of lipids in ALS and MND pathophysiology goes beyond energetic metabolism, to structure and signaling. These alterations, in particular those in the CNS, could represent an important source of therapeutic options in ALS. Indeed, there is a large spectrum of drugs targeting sphingolipids that are well characterized in terms of safety and bioavailability. They could be suitable for ALS patients, once clear targets are identified. A clinical trial has been very recently registered with the aim to test in ALS patients, the safety and tolerability of fingolimod, an agonist for sphingosine-1-phosphate receptors (NCT01786174), already used in multiple sclerosis to lower neuroinflammation in the CNS. In addition to pharmacological agents, dietary supplementation with neuroprotective and anti-inflammatory fatty acids, or derivatives, is worthy of further characterization. Altogether, alterations of lipid metabolism in MND deserve further work that will undoubtedly lead to new and interesting therapeutic options for patients.

### Conflict of interest statement

The authors declare that the research was conducted in the absence of any commercial or financial relationships that could be construed as a potential conflict of interest.
